# Sexual knowledge of Queensland forensic mental health patients: A cross-sectional quantitative study

**DOI:** 10.3389/fpsyt.2023.1160277

**Published:** 2023-04-11

**Authors:** Elnike Brand, Ching Tham, Angela Ratsch, Edward Heffernan

**Affiliations:** ^1^Faculty of Medicine, The University of Queensland, Brisbane, QLD, Australia; ^2^Metro South Hospital and Health Services, Queensland Health, Brisbane, QLD, Australia; ^3^Wide Bay Hospital and Health Service, Research Services, Hervey Bay Hospital, Hervey Bay, QLD, Australia; ^4^Rural Clinical School, The University of Queensland, Brisbane, QLD, Australia

**Keywords:** sexual knowledge, forensic, mental illness, mental health—related quality of life, sexual education

## Abstract

Forensic patients with serious mental illnesses (SMI) encounter significant challenges including active symptomatology, associated social and interpersonal impairments, psychotropic medication side effects and institutionalization, all of which negatively affect sexual functioning and possibly their acquirement of sexual knowledge. Evidence shows an increased prevalence of high-risk sexual behavior by this group however, there is an absence of literature examining the sexual knowledge of forensic patients. This quantitative cross-sectional study enrolled *N* = 50 patients currently under the treatment requirements of a Forensic Order and utilized the validated General Sexual Knowledge Questionnaire (GSKQ) to quantify the participants’ sexual knowledge over the domains of physiology, sexual intercourse, pregnancy, contraception, sexually transmitted diseases, and sexuality. Male forensic patients scored lower than females on all domains of sexual knowledge. All participants had reasonable knowledge of physiology, sexual intercourse and sexuality; of concern were poor scores on pregnancy, contraception and sexually transmitted diseases. Thirty-five (70%) of the respondents indicated that they had some limited sex education, mostly at school. Only six (12%) received any sexual education from a health professional despite extensive contact with the forensic mental health services across many years. There is a need to assess the deficit in sexual knowledge of forensic patients in order to develop sexual health education, intervention and rehabilitative programmes that cater to the specific needs of this group to improve their sexual knowledge, encourage safe and positive sexual experiences, and enhance their quality of life.

## 1. Introduction

This paper is part of a series of manuscripts in a Ph.D. study examining the sexual development, sexual health, sexual experiences, and sexual knowledge of forensic mental health patients, in Queensland, Australia (1).

Serious mental illnesses (SMI) are chronic and lifelong, affecting all aspects of a patient’s wellbeing (2). An important, but often overlooked aspect of wellbeing is sexual functioning (3). Significantly, active positive and negative symptomatology, associated social and interpersonal impairments, psychotropic medication side effects, and institutionalization negatively affect sexual functioning (4).

Within the category of “patients with a SMI” are those who are under the treatment requirements of a Forensic Order. These patients have committed a serious criminal offense, however, have been found of ‘unsound mind (insane)’ (5) at the time of the alleged offense, or “unfit for trial” (6). Evidence shows that for patients under the requirements of a Forensic Order, mental health illnesses mostly emerge in early life, in the teenage years, a time that coincides with acquiring sexual knowledge and first sexual experiences (7). Most of this population have also spent considerable time institutionalized (8) where they experience a lack of privacy, segregation, and severe restrictions (9, 10) and where they have diminished opportunities for sexual education and exposure to appropriate sexual behavior.

The sexual knowledge of several other populations with chronic and lifelong health challenges has been explored. For example, Lunsky et al. (11) suggested that for people diagnosed with an intellectual disability (ID):

The sexual development … is mitigated by a lack of normative learning experiences, segregation, imposed restrictions, lack of privacy, abuse, overuse of drugs which can inhibit or are administered to inhibit sexual drive, social attitudes that tend to infantilize individuals with ID or see them as sexually deviant, and a lack of knowledge about their own sexual rights.

An integrative literature review on the sexual healthcare needs of forensic patients demonstrated scant information or how health services manage these needs in inpatient patient or community settings (12).

It could be argued that patients on a Forensic Order are the most marginalized and stigmatized group within an already marginalized population. They face barriers similar to patients with an ID, with the additional challenges of risk mitigation and management of their SMI (9, 13, 14).

Quinn and Happell (10) and Hales et al. (15) both reported on a lack of sexual education for forensic patients and found that forensic patients had some awareness of sexual health risks but this was mostly of sexually transmitted diseases. In the study by Hales et al. (15), 68% (*n* = 17) of forensic inpatients could define “safe sex,” (specified as condom use) but like Quinn and Happell (10), Hales et al. (15) also concluded that few patients engaged in safe sex practices or used condoms.

The literature draws attention to a lack of comprehensive policies around sexual health within mental health settings (16–19) with several policies prohibiting or actively discouraging sexual expression and relationships (8, 20) based on risk aversion (9, 14) and restricted movement within facilities (21). Specifically, there is no consensus on what might constitute “best practice” in forensic settings (9, 22, 23) with inconsistencies across institutions and varied attitudes and management of sexual expression and an absence of patient-centered consideration for sexual healthcare needs (24). There is a dearth of evidence-based, individualized or group approaches that clinicians can utilize to assist forensic patients to achieve a healthy sexual life (12).

Given that there are few studies which have evaluated sexual development, functioning and sexual knowledge in people with SMI (25), and there is an absence of literature examining the sexual knowledge of forensic patients, the authors hypothesized that the sexual knowledge base of forensic mental health populations could be considerably lower than the general population and if so, that deficit could be addressed through education programs.

## 2. Aims

This study aims to quantify the sexual knowledge of forensic mental health patients. This information will aid in the identification of knowledge gaps, the development of specific sexual education strategies and tools, and intervention and rehabilitation programs which support and encourage safe and positive sexual experiences. This will ultimately contribute to enhancing the patient’s quality of life, and potentially increasing a positive engagement with health services and compliance with prescribed medications.

## 3. Research question

What is the sexual knowledge of people with a major mental illness who are treated under a Forensic Order?

## 4. Methodology

### 4.1. Study type and design

This paper reports the findings from Phase 1 (the foundation quantitative aspect) of a mixed-method, cross-sectional, prospective, face-to-face study conducted with Forensic Order patients in a regional area of South-Eastern Queensland in 2018. The findings reported here are a component of a larger study, the full design and method of which are reported elsewhere (1). At the time of the study, within the study area, the population of potential participants was *n* = 215. A review of the literature (26) showed 10 studies using a similar design, population and health survey with a mean sample size of 51 and seven studies having less than 25 participants. For this descriptive study, 50 completed surveys from this homogenous population was considered sufficient to address the research question and provide information for future studies (27).

### 4.2. Population of interest and sampling approach

The participant cohort were patients with SMI, who were mentally stable, community-based and treated under the requirements of a Forensic Order (hereafter known as forensic patients) in Queensland, Australia. Patients being treated under the requirements of a Forensic Order have a SMI diagnosis that has been confirmed by several psychiatrists and other mental health clinicians, and the diagnosis has been accepted by a court of law. A very structured and supportive clinical network is responsible for providing appropriate care and managing treatment to ensure and enable these patients to engage in recovery and rehabilitation. This team includes an authorized doctor, usually a forensic psychiatrist and a Forensic Liaison Officer (FLO). FLOs are registered clinicians including nurses, psychologists, and occupational therapists and have specialized training in mental health. The FLO leads the multidisciplinary mental health team and coordinates the holistic mental health care provided to forensic patients.

All forensic patients in the Wide Bay, Sunshine Coast and Metro North (MN) Hospital and Health Service (HHS) catchment areas (Table 1) were included as potential study participants. All patients that meet the inclusion criteria (Table 2) were considered eligible to participate. The index offense for which the Forensic Order was made was not considered during eligibility, recruitment, data collection or reporting. Potential participants who were judged by the FLO to be too unwell to participate (due to mental or physical health problems) or who could not consent, for example, those who have a cognitive impairment, were excluded. All participants with an intellectual disability (Full-scale < 70) were excluded from the study. All participants were proficient in the spoken English language. All participants had experienced severe mental illnesses, had previous periods of forensic inpatient admissions, and had been exposed to a multitude of psychotropic medications, including antipsychotics, antidepressants and mood stabilizers over prolonged periods.

**TABLE 1:** Number of patients treated under the requirements of a forensic order as of November 2017 in the study area.

South-East cluster adult mental health service	*N* =
Prince Charles (MN HHS)	59
Royal brisbane and women’s (MN HHS)	57
Redcliffe-caboolture (MN HHS)	35
Sunshine coast HHS	44
Wide bay HHS	19
Total	214

**TABLE 2:** Inclusion and exclusion criteria.

Inclusion criteria	Exclusion criteria
≥18 years	IQ < 70
Receiving treatment under the requirements of a Forensic Order Capacity to consent and participate Community-based	Vulnerable people (e.g., recent exposure or disclosure of sexual abuse or assault) Hospitalized for mental illness

### 4.3. Identification, enrollment, and informed consent

Forensic patients have very structured and supportive clinical networks coordinated by their forensic liaison officer (FLO) to ensure engagement in recovery and rehabilitation. The FLOs are the patient’s point of contact with Mental Health Services; they are highly familiar with the patient, and participate in regular clinical reviews, with each review dealing with patient’s sensitive and highly personal matters.

An arms-length approach to participant identification and initial recruitment occurred whereby the FLO considered each eligible patient and reviewed their circumstances at that point in time. If the FLO believed a patient was suitable and that the project was not a risk to the patient, the FLO then informed the patient of the research project. Patients indicated their agreement to contemplate enrollment by completing an *Approval to Provide Contact Information to Researchers* form. Only patients who had completed the form were reviewed for enrollment. A convenience sampling approach was used, with a target of 50 participants. Table 3 details the steps in the identification, enrollment and informed consent processes.

**TABLE 3:** Identification, enrollment, and informed consent processes.

Step 1	The research team liaised with the FLOs across the three HHS sites to discuss the project requirements. Information sessions were conducted with the FLOs to outline the research project and details, clarify inclusion and exclusion criteria, and discuss any concerns or issues.
Step 2	The initial screening process with no participant involvement occurred where the FLO identified potential exclusion participants (e.g., recent exposure or disclosure of sexual abuse, IQ < 70, and hospitalized patients).
Step 3	The FLO passed the research *Information Sheet* onto the identified potential participants who had at least 7 days to read the information and ask questions to the FLO. The FLO could contact the research team for any clarification in response to the questions.
Step 4	Potential participants who wished to be included returned the *Approval to Provide Contact Information to Researchers* form to the FLO.
Step 5	The FLO notified the researchers of potential participants, and the research team arranged contact where both the FLO and the potential participant were present for the consenting process.
Step 6	The research team member discussed the research with the potential participant and answered any additional questions.
Step 7	If the potential participant agreed to enroll, the formal *Participant Information and Consent* procedures, including the witness by the FLO, was conducted. It was explained to participants that they could choose to withdraw consent at any time during the interview and that in addition, the interviewer could decide to discontinue the interview process at any time.
Step 8	During the enrollment and consent phase, the research team members conducted an informal assessment with the focus on the participant’s capacity to consent and mental state. This was repeated before commencing each interview, and also during, and after each interview. The team members encouraged participants to discuss any concerns, distress, or discomfort with the interviewer at any time.
Step 9	An electronic format of the data collection tool was used. The interviewer read out the questions face-to-face to the respondent exactly as worded on the screen and then used colloquialisms, euphemisms, and explanations as required. The interviewer then entered the responses directly into the electronic questionnaires that automatically populated a de-identified password-secured, cloud-stored, electronic database. Participants were given the opportunity to have the questions explained or repeated if necessary and participants had the opportunity to have a short break during the interview process and to have a support person present for the duration of the interview.
Step 10	After the interview, the participant was debriefed by the trained interviewer and was given the opportunity to discuss any distress and discomfort that arose during the interview. The participants were also explicitly asked how distressing/uncomfortable they found the questionnaire.

### 4.4. Data collection: Interview process and data collection tool

Patients on a Forensic Order expect to and are obliged to have regular face-to-face contact with mental health services and a range of very personal information must be discussed at those interviews. The research team have never been involved in the patient’s clinical care. The research team considered that face-to-face interviews for this data collection technique provided the participants with skilled clinicians who could identify and respond immediately to any distress that arose during the interview.

This study utilized the validated General Sexual Knowledge Questionnaire (GSKQ) (28) to quantify the sexual knowledge of forensic patients across the domains of physiology, sexual intercourse, pregnancy, contraception, sexually transmitted diseases, and sexuality. The GSKQ is written to engage and capture the responses of a diverse population cohort, including those with and without ID (29), and those with limited communication skills, and basic education and has a maximum score of 110. These factors made it suitable for this participant population’s general lower level of education and literacy and vocabulary skills (1). The authors revised several items in the questionnaire to incorporate developments relating to contraception, as well as updating items that used outdated language. The GSKQ is easily administered, either by self or a clinician, and takes approximately 30 min to complete.

In this research, an electronic format of the GSKQ was completed by a research team member during the interview with each participant. The team was interested in recording the participant’s initial responses to the survey questions to prevent the risk of second interview process and recall bias, thus participants did not have the opportunity to review or edit their responses after their completion and the end of the interview.

### 4.5. Risk mitigation

The principal interviewer is a forensic psychiatrist who works clinically in the field of forensic psychiatry and has expertise in the assessment and management of all forensic patients. The remainder of the research team are qualified, skilled, and equipped to assess capacity to consent and the mental state of the participants both formally and informally, prior to and during the interview based on the participant’s presentation and answers to questions.

Given that SMI symptomatology may vary over time, the participants were screened throughout the recruitment phase and during and after the interviews, both subjectively and for any objective signs of distress or any signs and symptoms of emerging mental illness that might potentially influence their capacity to consent or participate in the research project. Specifically, the research team deliberated on the feedback from the FLO, the nature of the participants’ condition, their medication and treatment needs, the level of distress that they would be able to tolerate, and the complexity of the questions asked before, during and after consent and interview.

If the participant experienced significant distress, with or without other emerging symptoms of acute major mental illness, the local FLO was to be informed. The local FLO could then escalate any concerns to the local mental health treatment team. In case of major concerns regarding a participant’s mental state or risks, the research team were to arrange for an emergency assessment by the local acute care mental health teams following the HHS pathways for an emergency assessment of people suspected of experiencing a relapse of mental illness and increased risks to self and others. The FLO and treating teams were to be informed if this step had been implemented. In the event of an emergency assessment, an adverse research event would be lodged and discussed with the research team, including the research advisors.

### 4.6. Statistical analysis

Data was imported into SPSS^®^ version 20.0 (IBM^®^ SPSS^®^ Statistics 20) for Windows for analysis. Validation of the data accuracy following transfer was conducted by comparing the computed values on the Excel^®^ spreadsheets with the computed values in SPSS^®^. Within SPPS^®^, data were coded according to the tools’ analysis processes.

This is a descriptive study where descriptive analyses were undertaken of continuous and categorical variables with means ± SD or proportions reported for in-group dispersion and central tendency where applicable. Missing data and outliers are reported but excluded from the analysis. Outliers were identified using boxplots in SPSS^®^ and confirmed based on the established procedures from Hoaglin et al. (30).

## 5. Results

Fifty respondents consented, participated, and all completed the questionnaire. Table 4 shows that there were 43 males and 7 females. The participants’ ages ranged from 24 to 66 years old (mean age 41.2 years), (CI 95% –38.2 to 44.9 and CI 95% 32.7–45.9, respectively). Most of the respondents (88%) were born in Australia whilst 12% were born overseas (one each from New Zealand, Indonesia, Papua New Guinea, Sudan, the Philippines and France). All participants were proficient in the spoken English language with the majority (*n* = 47; 94%) speaking only English as their first language, and three participants also spoke one other language (Indonesian, French, and Sudanese, respectively). The majority were never married (80%), had no children (64%) and lived in the metro region (66%). Nearly half of the respondents identified with a religion (48%). Only six of the respondents had completed a university or college degree. Most respondents (94%) have worked at some time in the past, with 96% stating that they are unable to work at present. In terms of household income, 96% of the respondents earn less than $52,000 year. The survey took an average of 30 min to complete and there were no clinical indications of participant distress. All participants reported that the survey was not distressing and there was no escalation to the local mental health team for management of emerging symptoms of acute major mental illness.

**TABLE 4:** Demographic characteristics.

Respondents	Male *n* = 43	Female *n* = 7	Totals *N* = 50 (%)
**Age (years)**			
Mean	41.6	39.3	41.2
Range years	24–66	26–50	24–66
CI 95%	38.2–44.9	32.7–45.9	
**Country of birth**			
Australia	37	7	44 (88)
New Zealand	1		1 (2)
Indonesia	1		1 (2)
Papua New Guinea	1		1 (2)
Sudan	1		1 (2)
Philippines	1		1 (2)
France	1		1 (2)
**First language**			
English	40	7	47 (94)
Indonesian	1		1 (2)
French	1		1 (2)
Sudanese	1		1 (2)
**Legal marital status**			
Never married	36	4	40 (80)
Divorced	4	3	7 (14)
Separated	2	0	2 (4)
Civil/registered partnership	1	0	1 (2)
**Number of children**			
0 children	31	1	32 (64)
1 child	6	2	8 (16)
2 children	3	2	5 (10)
3 children	1	1	2 (4)
4 children	2	1	3 (6)
**Residence**			
Metro	29	4	33 (66)
Regional	14	3	17 (34)
**Religion**			
Yes	22	2	24 (48)
No	19	3	22 (44)
Maybe	2	2	4 (8)
**Qualifications**			
Secondary school	18	5	23 (48.9)
Trade certificate	5	0	5 (10.6)
Senior secondary school	11	2	13 (27.7)
Graduate certificate	3	0	3 (6.4)
Undergraduate university degree	3	0	3 (6.4)
**Past employment status**			
No (no paid employment in the past)	3	0	3 (6)
Yes (have been employed at some time)	40	7	47 (94)
**Current employment**			
Unemployed	0	1	1 (2)
Student	1	0	1 (2%)
Unable to work	42	6	48 (96)
**Family income before tax**			
<$28,000	41	4	45 (90)
28,000–$52,000	1	1	3 (6)
>$52,000	0	2	2 (4)

### 5.1. Sexual education

Sexual education was assessed by an initial screening question based on the GSKQ and no points were added to the data collection tool regardless of the answer. Out of the 50 respondents, 35 (70%) of the respondents indicated that they had some sex education from either school and/or their parents. Thirty-two (64%) of the respondents said that they had received sexual education at school whilst five (10%) had received it only through their parents.

Thirty-eight (76%) of the respondents reported that they had discussions of sex-related matters in the past with a combination of different groups of people. Thirty-one (62%) had discussed these matters with educational staff, only six (12%) with healthcare staff, and nine (18%) with their parents.

### 5.2. Physiology

Table 5 shows that all respondents could correctly name the different male anatomical parts on a male image, and most could correctly name the different female anatomical parts from the female image. However, one male did not know where the vagina was, and only 39 (78%) pointed to the correct anatomical site of the ovaries. The respondents generally provided correct responses to the questions related to puberty. Collectively, the respondents were able to name all the changes in boys and girls during puberty. There was a lesser understanding of questions about women’s ovaries and men’s sperm (ranging from 50 to 86%). The poorest answer was in whether women are born with their eggs or not, of which only 15 (30%) were able to answer correctly.

**TABLE 5:** Physiology questions.

	Group					
Questions	Male *N* = 43		Female *N* = 7		Total *N* = 50 (%)	
	Correct response		Correct response		Correct response	
Where are men’s sperm stored/kept?	38		5		43 (86)	
How many sperm might fit on a pinhead?	22		3		25 (50)	
Are men born with all their sperm, or are they made?	24		5		29 (58)	
What is puberty/adolescence?	43		7		50 (100)	
What age do boys reach puberty/adolescence?	41		7		48 (96)	
What is a period/monthly time of the month?	42		7		49 (98)*	
How old are women when they start their periods?	40		7		47 (94)	
How often do women have periods?	39		7		46 (92)	
Where are women’s eggs [ova] stored?	34		5		39 (78)	
How many ovaries are there?	30		6		36 (72)	
Are women born with all their eggs or are they made?	12		3		15 (30)	
	**Number of correct responses**	**Number of participants**	**Number of correct responses**	**Number of participants**	**Total number of correct responses**	**Total number of participants**
Can you name three things that happen to boys when they reach puberty (adolescence/teenage years/13–14)?	1 2 3	9 14 20	1 2 3	1 3 3	1 2 3	10 (20)** 17 (34)** 23 (46)**
Can you name three things that happen to girls when they reach puberty (adolescence/teenage years/13–14)?	1 2 3	5 17 19	–	1 2 3	1 2 3	5 (10)*** 20 (40)*** 23 (46)***

*A total of 45 participants (91.84%) indicated the discharge of blood and 4 (8%) indicated the change in the lining of the womb. **A total of 14 participants (28%) mentioned an increase in testosterone, 20 (40%) mentioned penis and scrotum enlargement, 10 (20%) said growth, with increased height and muscle and bones, 27 (82%) mentioned the change in voice, 41 (82%) mentioned the hair changes on the body and 2 (4%) mentioned puberty mood swings. ***A total of 33 participants (66%) mentioned the maturation of the uterus and ovaries, 37 (74%) the breast development, 31 (62%) mentioned the changes in hair, 6 (12%) mentioned mood swings and 7 (14%) stated the growth in height.

### 5.3. Sexual intercourse

Table 6 demonstrates that all respondents knew the meaning of “intercourse,” “erection” and “prostitute.” Almost all (98%) respondents knew what a virgin and masturbation were. The most poorly answered questions were about sexual intercourse during periods and the meaning of impotence, with only 60% correct responses.

**TABLE 6:** Sexual intercourse questions.

Sexual intercourse	Group		
	Male *N* = 43	Female *N* = 7	Total *N* = 50 (%)
	**Correct response**	**Correct response**	**Correct response**
Can you tell me what intercourse/having sex is?	43	7	50 (100%)
What is a virgin?	42	7	49 (98%)
Can you have intercourse/sex when a woman is having a period?	24	6	30 (60%)
What is an erection/hard-on?	43	7	50 (100%)
What is ejaculation/cumming?	40	7	47 (94%)
What is impotence?	25	5	30 (60%)
What is a prostitute/lady or man on the streets?	43	7	50 (100%)
What is incest?	39	6	45 (90%)
What is masturbation/wanking/jerking off/playing with self?	42	7	49 (98%)
Does masturbation/wanking/jerking off/playing with yourself have any negative/bad side effects on your health?	27	6	33 (66%)

### 5.4. Pregnancy

Information from Table 7 suggests that all respondents knew that pregnancy means a woman is going to have a baby and the part of a woman’s body that a baby comes out from. Less of the respondents (88%) were able to identify how a woman becomes pregnant, the time it takes to get pregnant and the purpose of the belly button. Collectively, the respondents managed to name nine of the ten indications that a woman is pregnant. The risk of pregnancy during the menstrual cycle was answered poorly with only 32 and 14% of the respondents identifying the answer.

**TABLE 7:** Pregnancy.

	Group					
Pregnancy	Male *N* = 43		Female *N* = 7		Total *N* = 50 (%)	
	Correct response		Correct response		Correct response	
Can you tell me what being pregnant means?	43		7		50 (100)	
How do women become pregnant?	Sex—42 IVF—14		Sex—7 IVF—3		49 (98) 17 (34)	
How long does it take from getting pregnant to having the baby?	39		7		46 (92)	
When is the risk of pregnancy least during a woman’s monthly cycle?	12		4		16 (32)	
From what part of a woman does the baby come out?	43		7		50 (100)	
What is the belly button/tummy button/navel?	37		7		44 (88)	
When is the risk of pregnancy highest during a woman’s monthly cycle?	5		2		7 (14)	
	**Number of correct responses**	**Number of participants**	**Number of correct responses**	**Number of participants**	**Total number of correct responses**	**Total number of participants**
How does a woman know that she is pregnant? (10 indications)	1 2 3 4 5	6 12 20 4 1	1 2 3 4 5	0 2 3 2 0	1 2 3 4 5	6 (12)* 14 (28)* 23 (46)* 6 (12)* 1 (2)*

*A total of 30 participants (60%) mentioned no periods, 19 (38%) nausea, 17 (34%) enlargement of the breasts, 10 (20%) hormonal changes, 4 (8%) carvings, 36 (72%) stomach size increase, 1 (2%) tiredness, 3 (6%) sense of knowing and 10 (20%) a positive pregnancy test.

### 5.5. Contraception

The results shown in Table 8 indicate that all respondents knew what contraception was but only two-thirds of respondents (66%) could name three types of contraception whilst only 48% could name three places to buy contraceptives. Only a small number of respondents could name additional forms of contraceptives (22%).

**TABLE 8:** Contraception.

	Group					
**Contraception**	**Male N = 43**		**Female N = 7**		**Total N = 50 (%)**	
	**Correct response**		**Correct response**		**Correct response**	
Can you tell me what contraception/birth control is?	43		7		50 (100)	
	**Number of correct responses**	**Number of participants**	**Number of correct responses**	**Number of participants**	**Total number of correct responses**	**Total number of participants**
Can you name three types of contraception?	1 2 3	3 12 28	1 2 3	0 2 5	1 2 3	3 (6)* 14 (28)* 33 (66)*
Can you tell me how a ——- works? (from 1 to 3 as above)	**	**	**	**	**	**
Can you name three places where you might buy/be given contraceptives?	1 2 3	8 15 20	1 2 3	0 3 4	1 2 3	8 (16)*** 18 (36)*** 24 (48)***
Can you name any other forms of contraception?	1 2 3 4	7 1 0 1	1 2 3 4	1 1 0 0	1 2 3 –	8 (16) 2 (4) 0 (0) 1 (2)

*A total of 41 participants (82%) mentioned a condom, 45 (90%) mentioned the contraceptive pill. 15 (30%) mentioned the contraceptive implant, 11 (22%) the diaphragm or cap, 4 (8%) the contraceptive long-acting injection, 3 (6%) the intra-uterine device, 3 (6%) sterilization, 3 (6%) mentioned withdrawal or coitus interruptus and 1 (2%) the natural rhythm method. **The result is based on the answer given to the prior question, 26 participants (52%) of the respondents could explain how the first method of contraception they mentioned worked, while out of the 47 respondents (25.53%) could explain how the second method they mentioned worked, and 12 out of the 33 (36.36%) respondents could explain how the third method worked. ***A total of 9 participants (18%) mentioned that you can get contraceptives at a hospital or health centre, 18 (36%) said at the general practitioner, 48 (96%) at the pharmacy or chemist, 36 (72%) said a shop or garage and 4 (8%) said at a family planning clinic.

### 5.6. Sexually transmitted diseases

Based on in-depth knowledge of the participant population (31) the researchers choose not to update the older term “sexually transmitted disease(s)” (STD) in the GSKQ survey tool (28) with the currently accepted and widely used term of “sexually transmitted infection(s)” (STI). Table 9 shows that 98% of the respondents knew what an STD was and 92% knew some contraceptives can prevent STDs. Only 64% of respondents could name three STDs and only 68% could name three symptoms of STDs. There was generally poorer knowledge of HIV with only 52% of respondents knowing what it was and only 18% knowing the difference between HIV and AIDS, although a majority (96%) knew at least one way someone might become infected with HIV.

**TABLE 9:** Sexually transmitted diseases.

	Group					
**Sexually transmitted diseases**	**Male *N* = 43**		**Female *N* = 7**		**Total *N* = 50 (%)**	
	**Correct response**		**Correct response**		**Correct response**	
What is an STD/STI/venereal disease?	42		7		49 (98)	
Which contraceptive can help prevent sexually transmitted diseases? (STD/VD/STI)	39		7		46 (92)	
What is HIV?	23		3		26 (52)	
What is the difference between HIV and AIDS?	9		0		9 (18)	
	**Number of correct responses**	**Number of participants**	**Number of correct responses**	**Number of participants**	**Total number of correct responses**	**Total number of participants**
Can you name five STDs?	1 2 3 4 5	6 8 13 9 4	1 2 3 4 5	1 3 1 1 1	1 2 3 4 5	7 (14)* 11 (22)* 14 (28)* 10 (20)* 5 (10)
Can you name any symptoms you might get if you had an STD? (from 1 to 3 as above)	1 2 3 4 5 6	5 10 14 5 1 1	1 2 3 4 5 6	1 3 1 1 1 0	1 2 3 4 5 6	6 (12) 13 (26) 15 (30) 6 (12) 2 (4) 1 (2)
How might someone become infected? with HIV?	1 2 3	14 28 0	1 2 3	1 5 0	1 2 3	15 (30)** 33 (66)** 0 (0)
How can you help protect yourself from the risk of getting HIV?	1 2 3	23 18 0	1 2 3	2 4 0	1 2 3	25 (50)*** 22 (44)*** 0 (0)

*A total of 23 participants (46%) mentioned Chlamydia, 21 (42%) Herpes, 32 (46%) Gonorrhea, 9 (18%) Syphilis, 30 (60%) HIV/AIDS, 6 (12%) genital warts, 3 (6%) Thrush, 4 (8%) Crabs and lice and 8 (16%) Hepatitis B and C. **A total of 48 participants (96%) knew it was through sexual intercourse and 33 (66%) also mentioned exposure to contaminated blood or blood products. None mentioned mother-to-baby transmission. ***A total of 47 participants (94%) correctly indicated condom use and 22 (44%) indicated the use of sterile needles.

### 5.7. Sexuality

There appears to be good knowledge regarding sexuality among the respondents with 98% of the respondents knowing the meaning of homosexuality, 80% knowing the meaning of heterosexuality and 80% knowing the meaning of bisexuality (Table 10).

**TABLE 10:** Sexuality.

	Group		
Sexuality	Male *N* = 43	Female *N* = 7	Total *N* = 50 (%)
	Correct response	Correct response	Correct response
What is a homosexual (Gay male/lesbian)?	42	7	49 (98)
What is a heterosexual (straight)?	33	7	40 (80)
What is a bisexual?	37	7	44 (88)

### 5.8. Overall scores

Table 11 shows the mean and standard deviation of the respondents’ scores for each section and total scores. The total score shows that the male respondents scored a mean of 67.2 (SD = 11.1) whilst the female respondents scored a mean of 69.9 (SD = 7.15).

**TABLE 11:** Means and standard deviations of the respondent’s scores on the General Sexual Knowledge Questionnaire (GSKQ) for both males and females.

	Group					
	Male			Female		
Section	Mean	SD	95% CI	Mean	SD	95% CI
1.(a) Physiology—pictures	15.7	0.492	15.6–15.9	15.9	0.378	15.6–16.1
(b) Physiology—Questions	13.3	3.08	12.3–14.2	13.7	2.06	12.2–15.2
2. Sexual intercourse	8.58	1.40	8.16–9.00	9.14	0.900	8.48–9.81
3. Pregnancy	8.07	1.78	7.54–8.60	9.14	1.95	7.70–10.6
4. Contraception	7.98	2.30	7.29–8.67	8.14	1.46	7.06–9.23
5. Sexually transmitted diseases	10.6	3.65	9.54–11.7	10.9	2.19	9.23–12.5
6. Sexuality	2.93	0.936	2.65–3.21	3.00	0.577	2.57–3.43
Total score	67.2	11.1	63.9–70.5	69.9	7.15	64.6–75.2

## 6. Discussion

This study conveniently enrolled and surveyed 50 community-based participants (43 men and seven women) from March to August 2020 who were under the requirements of a Forensic Order in Queensland. There was a disproportionate number of males compared to females in this study, however, this replicates the gender profile of Forensic Order (and prison) populations generally (32). It is difficult to compare the findings from the male forensic population to the female forensic population due to the small sample of females.

### 6.1. Demographics—The social determinants of mental health

The data highlights that most of the participants were middle-aged with a median age of 41.2 years. They were mostly single with no children, had completed secondary school, were unemployed, and were of low income. This profile is consistent with the literature regarding the adverse social determinants of health (WHO 2014). Compton and Shim (33) indicate that there are main “core” social determinants of mental health which include social exclusion, adverse early life experiences, poor education, unemployment, underemployment, job insecurity, poverty, income inequality, and neighborhood deprivation; poor access to sufficient healthy food; poor housing quality and housing instability; adverse features of the built environment and poor access to health care, that may lead to poorer mental health outcomes.

These factors appeared commonly in the participant population group. Although most of the participants were from urbanized areas where access to services such as mental health would be easier, they were more likely to be living in poorer neighborhoods, suffering from housing instability and had poorer access to food due to the cost and rent associated with living in an urbanized area (Note: the requirements of a Forensic Order require regular contact with the FLO which is more difficult if residing in more regional or remote locations, thus patients are required to live in more urbanized areas).

### 6.2. General Sexual Knowledge Questionnaire—What does the data show?

The GSKQ was developed by Talbot and Langdon (28) in the United Kingdom and is based on the knowledge areas in the Bender Sexual Knowledge Questionnaire (34). The GSKQ questionnaire consists of 63 items and six themes (physiology, sexual intercourse, pregnancy, contraception, sexually transmitted diseases, and sexuality). The use of the GSKQ in this study highlights some important sexual knowledge gaps for this population.

There was a deficit in formal sexual education, with 70% of the respondents indicating that they had some sex education, and the formal school system provided this education to only 64%. This was rather surprising given there is a sexual education curriculum in all Australian schools. In addition to this, 40% of the participants did not know what impotence was—this finding is particularly relevant as most forensic patients (and many mental health patients) are treated with psychotropics which cause impotence as a side effect (4). It is therefore important for a patient to understand what impotence means to communicate effectively with their treating practitioners about medication side effects.

Sexual dysfunction and psychopathology have a bidirectional influence on each other (35) and sexual dysfunction is a leading cause of poor quality of life in patients with SMI (36–44).

There is strong evidence to support the negative impact sexual dysfunction has on attitudes toward taking medication (40) and medication compliance (4, 37, 39, 44–53).

Although most participants knew what an STD was (98%) and which contraceptive could reduce the risk of STD (92%), there was a significant gap in the knowledge of participants in naming various types of STDs, the symptoms of STDs, risk of transmission and how to protect themselves. Only 44% knew that sterile needles could protect oneself from STDs and only 66% correctly mentioned transmission of STDs through contaminated blood. This finding is again of particular importance as many SMI patients have high rates of substance use and higher rates of STDs such as hepatitis B and HIV (7). Targeting the safe use of substances through an intravenous route and avoiding sharing of needles are significant areas that a sexual health education program in this population could have a lifelong impact.

### 6.3. Comparison data

The GSKQ is widely used in clinical practice to identify important sexual knowledge gaps in a range of populations, but with no emphasis on particular scores (29), therefore there is limited data published on the actual scoring of different populations to compare with the findings from this study. Two of the key authors in the field, Talbot and Langdon (28) used the GSKQ and provided data on sexual knowledge across four different groups (12 male sex offenders with ID who had engaged in treatment, 13 male sex offenders with ID who are yet to be offered treatment, 23 men and five women with ID with no sexual offending, and five men and five women without an ID and without a history of sexual offending).

The total scores for this study’s forensic male participants (mean 67.2 SD 11.1) and female participants (mean 69.9 SD 7.15) were higher than the total scores in the first three groups in Talbot’s study, (i.e., those diagnosed with ID). The survey participants scored lower compared with the fourth group in the Talbot study who had no ID and no history of sexual offending (mean 82.6 SD 7.88). In this study, the female participants scored higher in each section and had a higher total GSQK mean (69.9 SD 7.15) in comparison to the forensic male participants 67.2 (SD 11.1). However, they did score lower in all sections apart from sexuality (mean 3, SD 0.58) in comparison to the fourth group (no ID, no sex offending) in Talbot’s study 82.60 (SD 7.88). Jabareen and Zlotnick (54) used the GSKQ and concluded that boys and girls were similar in their low levels of sexual knowledge but differed by gender on the types of sexual knowledge. High school girls knew more about pregnancy, anatomy and physiology, while high school boys knew more about contraception.

This study shows a gap in the sexual knowledge of forensic mental health patients across all fields apart from the domain of sexuality that explored the terms: homo, bi and heterosexual. It is postulated that the sexuality domain results are superior in comparison to the Talbot and Langdon (28) results due to the more common use of these terms and acceptance of different gender and sexual identities in Australia in 2020 in comparison to the UK population in Talbot’s earlier study.

SMI patients have a higher prevalence of sexual dysfunction (4, 55) and high-risk sexual behaviors (56, 57). Nevertheless, the main finding of Quayle et al. (58) is that there were no significant differences in baseline sexual knowledge between tested sex offenders and non-sex offender patients detained in a high-security hospital in England. The sexual knowledge of both main groups of men appeared poor. Chaplin (59) however, did conclude that lower sexual knowledge is a risk factor in sexual offending.

Patients have identified the lack of opportunities to learn about sex, staff attitudes, lack of support and institutional rules as barriers to achieving their sexual health needs (60) and sexual education tended to produce a reduction in sexual risk behavior as opposed to complete cessation (61). The lack of basic professional sex education was identified as another barrier by McClure (16).

Risk-reduction interventions that target peer influence are advisable (62). It is of importance to address abuse trauma and victimization in interventions (18, 63–66) with education and training which explicitly recognize and attend to the complexity of sexual violence (67). Family and couple interventions, including relationship counseling and sex therapy (4, 68, 69) may be of value in supporting intimate relationships for patients with SMI (21, 70–72). There is a need for collaboration with health care practitioners (73) including general practitioners, sexual health, urological and gynecologic and obstetric services for people with mental illness (66) in order to prevent and treat sexually transmitted diseases including HIV/AIDS in people with mental illness (57, 63, 66, 74–76) including youth (77). Patients with severe mental illnesses, and their partners, may benefit from sex education to increase their knowledge about sex, sexual health, pregnancy, sexually transmitted diseases, and the use of contraceptives (4, 18, 21, 51, 55, 64, 78–82). Mental Health services should ensure the availability of contraceptives including condoms (83) and long-acting reversible methods (84). Patients should have access to psycho-education and information about sexual health programs (4, 42, 44, 50, 85, 86).

### 6.4. Poor sexual knowledge—Why?

The gap in sexual knowledge identified from this study is suggested to arise from several origins and influences. Lindstedt et al. (87) recognized that the impact of disruption from community life leads to stunted development and maturation, as well as cognitive and social difficulties—this disruption in the form of absence from schooling and hospitalization in this population early in their teenage years with the escalation of their mental health illness could possibly contribute to poor sexual knowledge. Females tend to develop SMI later in life which might explain the discrepancy between the male and female total scores in this study, with these women possibly having more opportunities and more exposure to sexual education during their early teenage years.

In addition, evidence shows that patients are reluctant to speak about their sexual health and knowledge unless approached (8, 13), and this was confirmed in this study with only six out of the fifty participants ever speaking to a healthcare practitioner about their sexual health. Compounding the patient’s reticence to discuss sexual health matters, is the awkwardness of clinicians around the theme of sexual health, with discussions on sexual health often being neglected and even avoided by clinicians (9). Research has shown that although clinicians recognize the importance of sexual health, there is an under-recognition and understanding of the need to explore sexual health and provide education (13, 88). Quinn and Happell (10) suggest that clinicians specifically lack awareness of their forensic patients’ sexual health, sexual function, sexual knowledge, and practices. Several reasons have been highlighted by clinicians, including the absence of sexual knowledge education strategies, lack of time, not being part of their “role,” or that it causes clinician discomfort and anxiety (14).

Deepening the gap is that institutionally, forensic mental health management prioritizes risk mitigation and mental health symptom reduction to the extent that there is a vacuum in addressing sexual health as part of holistic care. This failure is in contradiction to the role of mental health services to support human rights and has a negative impact on a patient’s emotional and sexual wellbeing (89) and Brand et al. (1) confirms that forensic patients often lack knowledge regarding their sexual rights. This systemic barrier has resulted in the absence of comprehensive treatment programmes to address the sexual health and knowledge of SMI patients in general, and specifically in the forensic setting.

A few reviews have attempted to study a range of sexual health risk reduction interventions for people with SMI (22, 61, 90), and an Australian study by Quinn et al. (91) demonstrated that the implementation of a brief education program about sexual issues of patients in mental health services can result in sustained patient behavioral change. However, no clear guidelines or recommendations exist regarding the improvement of sexual knowledge in forensic patients.

### 6.5. Implications of poor sexual knowledge

Sexual health and knowledge form an important part of the human being (92) and general sexual knowledge plays a key role in romantic relationships, intimacy, and sexuality in all populations, and is recognized as a component of recovery in mental health illness (93). Despite widespread national, state, and local investment targeted at reducing stigma and improving access and support for all aspects of health care (94), patients with SMI are marginalized and stigmatized (95). SMI patients have a higher prevalence of sexual dysfunction (4, 55) and high-risk sexual behaviors (56, 57). For example, both Dickerson et al. (66) and Just (40) show that poor sexual health in SMI patients negatively impacts their relationships, and emotional and physical wellbeing and reduces their quality of life (38, 40, 41) and adherence to psychotropic medications (96). On the other hand, sexual health education reduces sexual risk behavior (61) with Yeung et al. (97) confirming a strong association between sex education and using contraception at first vaginal intercourse [odds ratio (OR) = 1.57; 95% confidence interval (CI) 1.44–1.71; *P* < 0.001] and higher levels of STI knowledge (OR = 1.75; 95% CI 1.46–2.12; *P* < 0.001) in the general Australian population.

## 7. Limitations

This study considers the responses from 50 forensic mental health participants in Queensland. Less women were enrolled in the study, however, the number mimics the normal gender profile of forensic patients. The study was limited by the nature of the participants’ mental health symptoms and medication which impacted their potential inclusion and potentially impacted on their communication ability and skills. GSKQ (28) assesses sexual knowledge and not social skills relating to sexual encounters (for example, courtship), thus the important social support factors related to sexual health such as the role of education, family, culture or religion and their impact on the sexual knowledge of the participants were not explored in this study. In addition, the study did not consider the length(s) of institutionalization or the nature of the participant’s index offense(s).

Australian, and specifically Queensland forensic mental health services adhere to a strict no-sex policy and would not allow inpatients access to sexual relationships, pornography, etc. The authors acknowledge that patients in settings with more liberal policies would have potentially scored differently.

## 8. Recommendations

Most forensic patients will eventually be transferred from institutions and acute services to the community (98), although, according to a study on Swedish forensic male patients, 73% will require ongoing support to enable sustained community living (87). It is important to have a clear understanding of the sexual knowledge of forensic patients to identify areas where this already vulnerable population can be supported to achieve safe and healthy sexual health through appropriate sexual education, intervention, and rehabilitation programs that encourage safe and positive sexual experiences, enhance their quality of life and promote sexual wellbeing. Forensic patients should have access to psychoeducation and information about sexual health programs (4, 42). These patients, and their partners, will likely benefit from sex education to increase their knowledge about sex, sexual health, pregnancy, sexually transmitted diseases, and use of contraceptives (55, 78, 99).

Sexual education should be introduced and promoted as part of holistic mental health intervention, from the early stages of the recognition of symptoms of SMI (100) and should incorporate education around dating expectations and etiquette and social and relationship-building skills training (21, 93). The most effective sex education might be achieved in gender-neutral groups where patients practice social and communication skills (8, 85, 86) and involve practical skills training (82, 86). However, Hajagos et al. (101) recommended an individual rather than a group teaching approach to the sensitive subject of safer sex. Safer-sex education for SMI patients must include repetitive, interactive education capitalizing on verbal, visual, written, tactile, and motor skill teaching methods to compensate for potential cognitive deficits (101). This education could be conducted through support groups. Support and rehabilitation groups exist for a range of other health needs (for example drug and alcohol, domestic violence, and dementia), however, support structures are absent for forensic patients to obtain sexual education and knowledge (22) and it is recommended that such groups be introduced.

Forensic mental health clinicians are skilled in the field of forensic mental health and are tasked to support forensic patients with their psychosocial needs, recovery and rehabilitation. However, the exploration of a patient’s sexual health and sexual knowledge by clinicians appears to be lacking (12, 31). Review and management of sexual health and knowledge should be a standardized part of a mental health practice (102) as it is an essential component of overall wellbeing. Given that sexual knowledge can be easily taught, sexual education needs to become a priority in mental health clinician education, preparation and professional development (103).

## 9. Conclusion

This study aligned with the Queensland Sexual Health Strategy (104). The Strategy aims to improve the sexual and reproductive health of all Queenslanders by addressing a broad range of sexual and reproductive health issues including sexual health promotion, prevention, clinical service provision and community education. Moreover, this study aligned with the recovery principles in mental health (105).

From the perspective of the individual with mental illness, recovery means gaining and retaining hope, understanding of one’s abilities and disabilities, engagement in active life, personal autonomy, social identity, meaning and purpose in life, and a positive sense of self (106). This includes the enhancement of knowledge in the important field of sexuality with the hope that this knowledge will aid in encouraging safe and fulfilling sexual experiences.

## Data availability statement

The raw data supporting the conclusions of this article will be made available by the authors, without undue reservation.

## Ethics statement

The studies involving human participants were reviewed and approved by Ethical and scientific review has been obtained from the Human Research Ethics Committees (HREC) at the Royal Brisbane and Women’s Hospital (HREC/18/QRBW/56) and the University of Queensland. The patients/participants provided their written informed consent to participate in this study.

## Author contributions

EB with assistance from CT conceived and developed the method and undertook the data collection for this research. CT involved in the interpretation of the qualitative analysis. EB and CT wrote the initial manuscript. AR and EH edited the manuscript. All authors have approved the final manuscript.
